# Online Detection System for Wheat Machine Harvesting Impurity Rate Based on DeepLabV3+

**DOI:** 10.3390/s22197627

**Published:** 2022-10-08

**Authors:** Man Chen, Chengqian Jin, Youliang Ni, Jinshan Xu, Tengxiang Yang

**Affiliations:** Nanjing Institute of Agricultural Mechanization, Ministry of Agriculture and Rural Affairs, Nanjing 210014, China

**Keywords:** wheat, impurity rate, dynamic detection, image segmentation, DeepLabV3+

## Abstract

Wheat, one of the most important food crops in the world, is usually harvested mechanically by combine harvesters. The impurity rate is one of the most important indicators of the quality of wheat obtained by mechanized harvesting. To realize the online detection of the impurity rate in the mechanized harvesting process of wheat, a vision system based on the DeepLabV3+ model of deep learning for identifying and segmenting wheat grains and impurities was designed in this study. The DeepLabV3+ model construction considered the four backbones of MobileNetV2, Xception-65, ResNet-50, and ResNet-101 for training. The optimal DeepLabV3+ model was determined through the accuracy rate, comprehensive evaluation index, and average intersection ratio. On this basis, an online detection method of measuring the wheat impurity rate in mechanized harvesting based on image information was constructed. The model realized the online detection of the wheat impurity rate. The test results showed that ResNet-50 had the best recognition and segmentation performance; the accuracy rate of grain identification was 86.86%; the comprehensive evaluation index was 83.63%; the intersection ratio was 0.7186; the accuracy rate of impurity identification was 89.91%; the comprehensive evaluation index was 87.18%; the intersection ratio was 0.7717; and the average intersection ratio was 0.7457. In terms of speed, ResNet-50 had a fast segmentation speed of 256 ms per image. Therefore, in this study, ResNet-50 was selected as the backbone network for DeepLabV3+ to carry out the identification and segmentation of mechanically harvested wheat grains and impurity components. Based on the manual inspection results, the maximum absolute error of the device impurity rate detection in the bench test was 0.2%, and the largest relative error was 17.34%; the maximum absolute error of the device impurity rate detection in the field test was 0.06%; and the largest relative error was 13.78%. This study provides a real-time method for impurity rate measurement in wheat mechanized harvesting.

## 1. Introduction

### 1.1. Background

Wheat is one of the most important food crops in the world. The sown area and output of wheat rank first among all food crops. One third of the world’s population depends on wheat as their staple food. At present, the harvesting method for wheat is generally mechanized harvesting using a combine harvester at the mature stage. By 2021, the level of mechanized wheat harvesting in China had reached 97.49% [1]. The impurity rate is an important indicator of the quality of wheat mechanized harvesting. The impurity rate is the percentage of impurities in wheat harvested mechanically. Impurities refer to non-grain substances such as wheat straw and awn. However, if the parameters of the combine harvester are improperly set, the impurity content of mechanically harvested wheat will be too high [2]. Existing combine harvesters generally lack an online detection system for impurity rate and thus cannot provide drivers with real-time harvesting information; this lack of information affects the quality of mechanically harvested wheat [3]. Therefore, it is important to realize online detection of the impurity rate of wheat in mechanized harvesting.

The common methods for the determination of the impurity rate in wheat mechanized harvesting are manual visual inspection and sampling inspection. Manual visual inspection involves the driver observing the harvested grains in the granary through the observation window on the combine harvester and qualitatively judging the impurity rate of the wheat. For manual sampling and testing, it is necessary to stop the machine to sample from the grain tank and manually separate the grains and impurities in the wheat samples to obtain the impurity rate after weighing. However, these two methods rely on human judgment, and hence are error-prone, time-consuming, and unable to provide real-time impurity rate information. It is important to quickly and accurately determine the impurity rate of mechanically harvested wheat. In recent years, machine vision and image processing technology have played key roles in the grain quality inspection of soybeans for impurities and broken grains [4], detection of rice impurities and broken grains [5], detection of impurities in seed cotton [6], and classification of wheat varieties [7,8]. The technology has the advantages of rapid detection and online measurement, factors that make up for the shortcomings of traditional detection methods. However, machine vision and image processing algorithms have problems such as severe over-segmentation, segmentation parameters relying on human experience, and requirement of large image sets. The time required for detection makes it difficult to meet the actual requirements of mechanized production [9].

### 1.2. Literature Review

With the rapid development of deep learning, machine vision technology integrated with deep learning is a rapidly emerging nondestructive detection method that not only contains the image information of the target to be detected but also can obtain richer feature information from a small data set. This improves the segmentation accuracy of the target image to be detected [10]. Machine vision technology incorporating deep learning has been widely used in the examination of grain target features such as grain damage [11], wheat high-throughput yield phenotyping [12], wheat variety Identification [13], and individual tree detection and species classification [14]. Semantic segmentation is a very important direction, as it enables image pixel-level classification. DeepLabV3+ is a typical semantic segmentation network. In order to integrate multi-scale information, it introduces the resolution of features extracted by the encoder that can be arbitrarily controlled and balances accuracy and time consumption through atrous convolution.

At present, DeepLabV3+ is widely used in grain target detection. For example, Zhao et al. realized the segmentation and counting of rapeseed [15]; Zhang et al. realized the automatic extraction of wheat lodging area [16]; Bhagat et al. realized the plant leaf segmentation and counting [17]; and Yang et al. achieved the efficient segmentation of soybean planting areas [18].

Although the DeepLabV3+ technology is now widely used, there are no relevant reports concerning the application of DeepLabV3+ to the detection of the impurity rate of mechanically harvested wheat. Shen et al. achieved fast and effective detection of impurities in wheat based on terahertz spectral imaging and a convolutional neural network, but that study used a convolutional neutral network to extract data and information of sample composition characteristics [19]. Chen et al. used the least squares support vector machine to construct an inversion model of the wheat sample impurity rate based on different indicators, but this technology cannot be applied to the detection of wheat impurities in the mechanized harvesting process [20]. In the above reports, the detection of wheat impurities was completed in the laboratory, and there remained a certain gap between laboratory and practical application.

DeepLabV3+ has both encoder and decoder modules. In the encoder module, feature maps are obtained through the backbone feature extraction network. MobileNetV2, Xception, and ResNet are often used as backbone feature extraction networks. The effect of each backbone feature extraction varies according to different detection targets. Wu et al. found that using ResNet-101 to segment abnormal leaves of hydroponic lettuce was the best, while ResNet-50 demonstrated a high segmentation speed [21]. Sun et al. constructed a band information enhancement (BIE) module and proposed a DeepLabV3+ grape-growing area identification method with enhanced band information [22]. This method segmented the grape-growing area more completely and showed a good edge recognition effect. Mu et al. found that the use of ResNet-101 could accurately identify rice lodging, and the accuracy of rice lodging image recognition was 0.99 [23]. Dai et al. found that the use of MobileNetV2 could quickly and effectively monitor the occurrence of wheat scab, and the average accuracy of the model was 0.9692 [24]. Based on the above analysis, DeepLabv3+ could in theory encode rich contextual information and use a simple and effective decoder module to recover object boundaries; this could capture multi-scale information and effectively utilize the detailed information of the image and the spatial correlation of pixels in a large range. Selecting different backbone feature extraction networks resulted in detailed differences in the performance of DeepLabv3+. These studies have provided new ideas for the application of DeepLabv3+ regarding the online detection of wheat impurity rate in mechanized harvesting.

### 1.3. Contributions

This study explored the feasibility of detecting the impurity content of mechanically harvested wheat based on machine vision and deep learning technology. MobileNetV2, Xception-65, ResNet-50, and ResNet-101 were adopted as candidate backbone feature extraction networks for the DeepLabv3+ model. Through comparisons of the modeling effects of different backbone feature extraction networks by comprehensive evaluation indicators, average cross-combination ratio, and image processing speed, the optimal recognition and segmentation model was finally determined. Finally, the optimal DeepLabv3+ model was used to construct an online detection algorithm for the mechanized harvesting impurity rate based on image information, and the feasibility and accuracy of the algorithm were verified by experiments.

## 2. Materials and Methods

### 2.1. Online Detection Device for Wheat Impurity Rate

**Figure 1:**
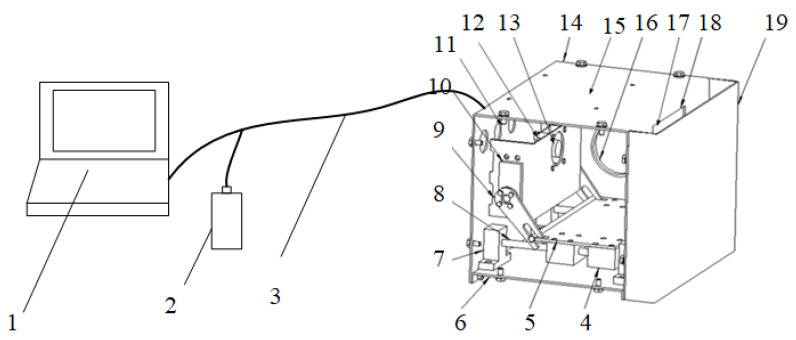
Online detection device for wheat impurity content in mechanized harvesting. (1 computer; 2 12-V DC power supply; 3 system bus; 4 sliders; 5 telescopic plates; 6 bases; 7 rail seats; 8 rails; 9 levers; 10 DC servos; 11 data bus connectors; 12 industrial cameras; 13 Industrial camera fixing bracket; 14 shell; 15 embedded data processing module; 16 LED visual light source; 17 transparent plexiglass; 18 photo window; 19 sampling chamber.)

We developed an online device for detection of the impurity rate of mechanically harvested wheat. The device included an Ubuntu 20.04 host, a 12-V DC power supply, an industrial camera, a servo, and other parts (Figure 1). The device contained an industrial camera (LRCP10230, SinoVision, Guangzhou, China) to acquire images of wheat samples. The industrial camera was set facing the photo window of the sampling bin, with a lens with a focal length of 12 mm, and the lens was 105 mm away from the transparent plexiglass. Under the LED visual light source, the RGB (red, green, blue) wheat sample images captured by the industrial camera had a resolution of 1280 pixels × 1024 pixels and were saved in JPEG format. The device also included two DC servos (LX-20, Magic Technology, Shenzhen, China). By controlling the forward and reverse rotation of the DC servos, a telescopic plate could be retracted or extended to realize the dynamic updating of the wheat in the sampling bin.

The device requires an Ubuntu 20.04 host (Tsinghua Tongfang T45PRO laptop, Tongfang Co., Ltd., Wuxi, China, Intel^®^ Core^®^ i7-6500U processor, 16 GB DDR4 3200 MHz memory, and 6 GB Nvidia GeForce RTX3060 graphics card). The Ubuntu 20.04 host was primarily used to run the online detection algorithm of wheat impurity content. The machine acquired images of wheat samples via USB-controlled industrial cameras. The recognition and segmentation of wheat samples was realized by running the DeepLabv3+ model. After obtaining the number of pixels of grains and impurities in the image, the impurity rate of the sample to be tested was calculated through a quantitative model.

### 2.2. The Network Architecture of DeepLabV3+

**Figure 2:**
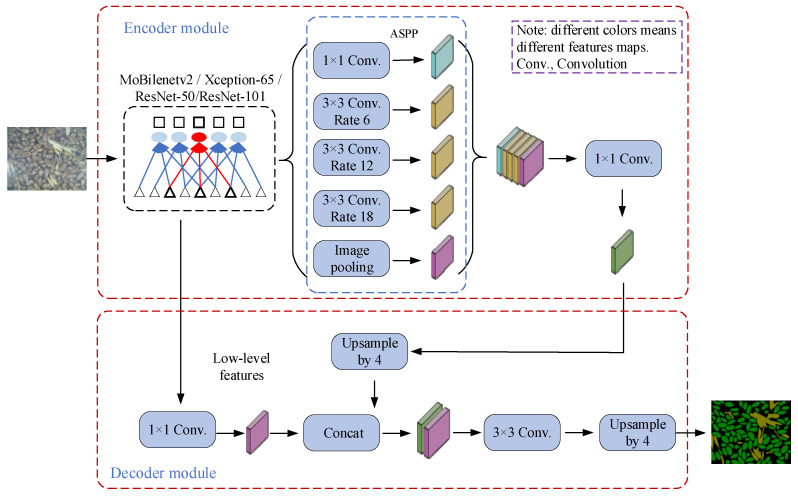
Block diagram of the action of DeepLabV3+ on mechanized harvested wheat images.

The DeepLabv3+ network was proposed in 2018 [25] and was an improvement on the original DeepLabv3 network. This version is currently the best performing network in the DeepLab network series. The network structure was divided into an encoder and a decoder, as shown in Figure 2.

In the upper part of the encoder (Figure 2), MobileNet, ResNet, and Xception could be selected as the backbone network. In order to make the extracted features have a larger receptive field, normal convolution was replaced with dilated convolution in the last coding block. The atrous spatial pyramid pooling model composed of atrous convolution with different expansion coefficients was used to encode the image context information and splicing and fusion. Then, we used 1 × 1 convolution to adjust the number of output channels to improve the generalization ability of network feature extraction.

The lower part of the decoder used bilinear interpolation to upsample the feature tensor output from the encoder by a factor of 4. Then, it was spliced with the feature map of the corresponding level of the backbone network, and the detailed information carried by the shallow features was captured by the cross-layer connection in order to enrich the semantic information and detailed information of the image. Finally, the fused feature map was upsampled four times to obtain a semantic segmentation map with the same size as the original image.

To develop a better mechanized harvested wheat image segmentation model, we trained four backbone networks, namely MobileNetV2, Xception-65, ResNet-50, and ResNet-101, based on DeepLabV3+.

### 2.3. DeepLabV3+ Network Training

#### 2.3.1. Data Annotation and Augmentation

In order to train a wheat component segmentation model for mechanized harvesting based on DeepLabV3+, a total of 500 wheat images with a resolution of 1280 pixels × 1024 pixels were collected as the original dataset in this study.

**Figure 3:**
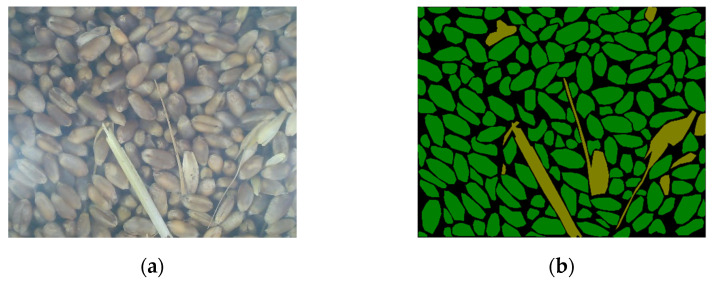
An example RGB image of wheat samples in the photo frame of the wheat mechanized harvesting online detection device (**a**) and the wheat sample labelled image with “polygons” (**b**). Black polygons, green polygons, and beige polygons represent areas marked as “background,” “grain,” and “impurity,” respectively.

We used LabelMe (Version No. 3.16.7, Massachusetts Institute of Technology, Cambridge, MA, USA) to label the wheat image dataset, to label wheat grains and impurities with polygons, and then assigned labels, where the background was labeled as 0, grains were labeled as 1, and impurities were labeled as 2. In order to reduce the computational complexity of the model and improve the detection time, each image was scaled to 512 pixels × 512 pixels through bilinear interpolation. Examples of RGB images and their corresponding label images are shown in Figure 3a and Figure 3b, respectively.

To avoid unbalanced performance evaluation on the test set, the datasets were randomly selected as a training set (350 images), a validation set (50 images), and a test set (100 images) in a ratio of 7:2:1. Data augmentation played a crucial role in the training of deep learning models. To improve the robustness of the model, data augmentation was performed on the limited dataset. Images in the training and validation sets were subjected to 90° and 270° image counterclockwise rotation, 0.6× and 1.8× image scaling, and image mirroring on the horizontal and vertical axes. After data augmentation, the training set consisted of 2450 images, and the validation set consisted of 350 images. The test set was unaugmented and consisted of 100 images.

#### 2.3.2. Network Training

DeepLabV3+ models were trained and tested using an Ubuntu 20.04 host (Dell Precision 7920 Tower graphics workstation, Dell, Xiamen, China) with GPU (Nvidia Quadro RTX5000 16 GB GPU), 26-core CPU (dual Intel^®^ Xeon^®^ Gold 6230R, 4.00 GHz) and 128 GB DDR4 3200 MHz memory.

Deployed models were based on Python 3.6, torch 1.2.0, torchvision 0.4.0, scipy 1.2.1, numpy 1.17.0, matplotlib 3.1.2, opencv_python 4.1.2.30, tqdm 4.60.0, Pillow 8.2.0, h5py 2.10.0 The training environment used GPU and CPU dual devices to train and test different networks.

In this study, to train the DeepLabV3+ model on the wheat image dataset, the weights of the pretrained model were used to initialize and fine-tune the model through further training. These initial weights were obtained from pretrained models on the PASCAL VOC 2007 dataset [26].

### 2.4. Test Design

#### 2.4.1. Bench Test Design

**Figure 4:**
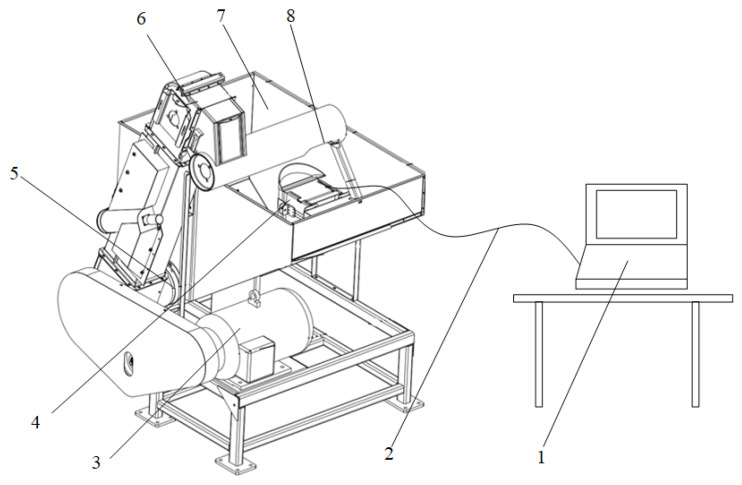
Bench verification test. (1 computer; 2 system bus; 3 motor; 4 wheat impurity rate detection device; 5 auger; 6 scraper elevator; 7 grain tank; 8 discharge hopper.)

In order to test the performance of the online detection device for the impurity rate of wheat harvested by mechanization, an indoor test bench was constructed in this study. The test bench consisted of a rack, a grain tank, a scraper elevator, a motor, and a wheat impurity rate detection device, as shown in Figure 4.

When the bench was working, the motor drove the auger to rotate, and the wheat in the grain tank was transferred to the scraper elevator. There was a hopper at the top of the elevator. During the process of dropping the wheat from the hopper into the grain tank, part of the wheat entered the sampling bin of the wheat impurity rate detection device. After the detection device completed the detection of the wheat samples, the wheat in the device fell back into the grain tank.

A total of three batches of wheat samples were prepared for the bench test, and repeated tests were carried out. The wheat was collected from an experimental field in Daba Village, Yongchang County, Jinchang City, Gansu Province. The wheat variety was Longchun 39; the moisture content was 12.4%, and the thousand-kernel weight was 46.87 g. When testing the impurity rate of each batch of wheat, referring to DG-T 014-2019 “Grain Combine Harvester,” three wheat samples (500 g each) were randomly selected manually to estimate the impurity rate of the samples. Then, the test bench motor was run, and the wheat impurity rate detection device dynamically sampled and detected the wheat 30 times and automatically recorded the test data. Finally, the results of the wheat impurity rate detection device and manual detection were analyzed and compared, and the online detection effect of the wheat impurity rate based on the DeepLabV3+ model was verified.

#### 2.4.2. Field Trial Design

In order to verify the detection effect of the mechanized harvesting wheat impurity rate online detection device during the field harvesting process, we installed the devise on a combine harvester (Wode Ruilong Zhihang version combine harvester, Model 4LZ-7.0EN(Q)) and carried out field test experiments. The study site was an experimental wheat field in Daba Village, Yongchang County, Jinchang City, Gansu Province. The wheat variety was Longchun 39; the moisture content was 12.3%, and the thousand-kernel weight was 47.05 g. The test date was 24 July 2022.

**Figure 5:**
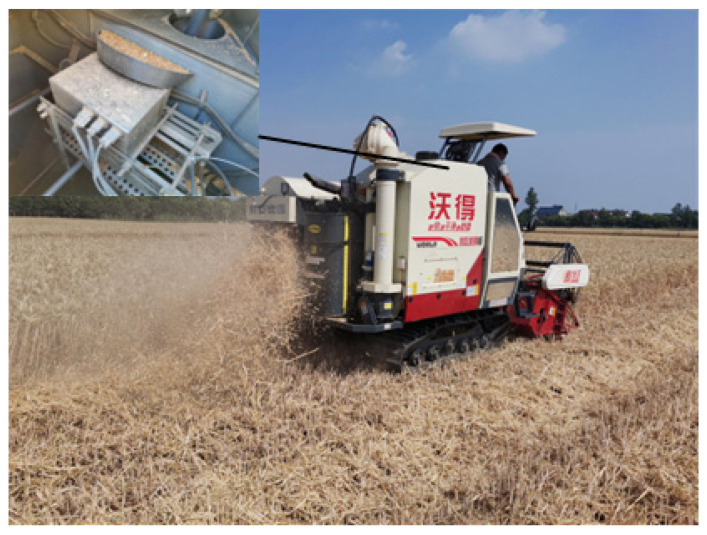
Field validation experiment. (Note: the combine harvester (Wode Ruilong Zhihang version combine harvester, Model 4LZ-7.0EN(Q)), FMWORLD, Danyang, China).

This field test comprised a repeated test of three trips, with a single trip length of 200 m and an operating speed of 4 km/h. The test site is shown in Figure 5. The impurity rate detection device was installed below the grain outlet of the combine harvester, connected to the notebook through a data bus, and powered by a 12-V DC battery.

During each test, the online device automatically detected the real-time impurity rate of the wheat during the harvesting operation and recorded the test data. Then, the harvester was stopped to unload the grain; the wheat in the grain tank was emptied; three random samples were taken manually; and the impurity rate of the wheat in this trip was obtained by testing. Finally, the test data were used to analyze the performance of the online detection device for estimating the wheat impurity rate.

### 2.5. Performance Evaluation

#### 2.5.1. Network Recognition and Segmentation Performance Evaluation Index

In this study, the precision rate *P*, the recall rate *R*, the comprehensive evaluation index *F*_1_, the intersection ratio *F*_IOU_, the average intersection ratio *F*_MIOU_, and the average processing speed *I*_v_ of a machine-harvested wheat sample image were used as evaluation indicators of the image recognition and classification results of different models, and they were calculated as follows:

(1) $$P=\frac{T_{P}}{\left(T_{P}+F_{P}\right)},$$

(2) $$R=\frac{T_{P}}{\left(T_{P}+F_{N}\right)},$$

(3) $$F_{1}=\frac{2\times P\times R}{P+R},$$

(4) $$F_{\mathrm{IOU}}=\frac{T_{P}}{\left(T_{P}+F_{N}+F_{P}\right)},\;\mathrm{and}$$

(5) $$F_{\mathrm{MIOU}}=\sum_{i=1}^{n}\frac{F_{{\mathrm{IOU}}_{i}}}{n},$$

where *P* represents the precision rate; *R* represents the recall rate; *F*_1_ represents the comprehensive evaluation index; *T*_P_ represents the number of correctly classified pixels predicted; *F*_P_ represents the wrongly classified pixels predicted; *F*_N_ represents the correctly classified pixels predicted to be misclassified pixels; *n* represents the number of categories of the classification; *F*_IOU_ represents the intersection ratio; *F*_MIOU_ represents the average intersection ratio, and *I*_v_ represents a machine-harvested wheat sample average image processing speed in ms.

#### 2.5.2. Performance Evaluation of Wheat Impurity Content Detection Based on Image Information

In the existing methods for detecting the impurity rate of a wheat combine harvester the impurity rate is the percentage of the mass of the grains made up by the mass of impurities in the sample. According to the existing measurement methods, a quantification model of pixel-based impurity rate was formulated. The calculation formula was:

(6) $$P_{cz}=\frac{w_{z}}{w}\times 100\%,$$

(7) $$P_{z}=\frac{T_{z}}{\left(T_{z}+\partial T_{w}\right)}\times 100\%,$$

where *P*_cz_ represents the manual measurement of impurity rate in percent; *w*_z_ represents the mass of non-grain substances in manually sampled samples in gram; *w* represents the mass of manually sampled wheat samples in gram; *P*_z_ represents the impurity rate in percent; *T*_w_ represents the number of pixels of grains in the predicted image; *T*_z_ represents the number of impurities pixels in the predicted image; *∂* represents the ratio of the average mass of grains to the average mass of impurities at 1000 pixel points. Under laboratory conditions, the value of *∂* was 11.8906 by manual calibration.

The coefficient of variation, the absolute error, and relative error between the average value of system detection and manual detection results were used to evaluate the effect of online monitoring of wheat machine harvesting impurity rate based on DeepLabV3+. The calculation formulas were as follows:

(8) $$R_{\mathrm{az}}=\left|\bar{P_{\mathrm{Sz}}}-\bar{P_{\mathrm{Mz}}}\right|,$$

(9) $$R_{\mathrm{rz}}=\frac{\left|\bar{P_{\mathrm{Sz}}}-\bar{P_{\mathrm{Mz}}}\right|}{\bar{P_{\mathrm{Mz}}}}\times 100\%,$$

(10) $$R_{\mathrm{Scv}}=\frac{\sqrt{\frac{1}{N-1}\sum_{i=1}^{N}{\left(P_{\mathrm{Sz}i}-\bar{P_{\mathrm{Sz}}}\right)}^{2}}}{\bar{P_{\mathrm{Sz}}}}\times 100\%,\;\mathrm{and}$$

(11) $$R_{\mathrm{Mcv}}=\frac{\sqrt{\frac{1}{N-1}\sum_{i=1}^{N}{\left(P_{\mathrm{Mz}i}-\bar{P_{\mathrm{Mz}}}\right)}^{2}}}{\bar{P_{\mathrm{Mz}}}}\times 100\%,$$

where $\bar{P_{\mathrm{Sz}}}$ represents the average impurity rate of samples detected by the system in percent; $\bar{P_{\mathrm{Mz}}}$ represents the average value of impurity rate of samples detected manually in percent; *R*_az_ represents the absolute error of impurity rate in percent; *R*_rz_ represents the relative error of impurity rate in percent; *R*_Scv_ represents the coefficient of variation of the device detection value in percent, and *R*_Mcv_ represents the variation coefficient of the manual detection value in percent.

## 3. Results and Discussion

### 3.1. Comparison of Different Backbones

In terms of grain recognition and segmentation accuracy, MobileNetV2, Xception-65, ResNet-50, and ResNet-101 achieved 85.31%, 83.57%, 86.86%, and 84.54% similar *P*, as shown in Table 1. The DeepLabV3+ model with Xception-65 outperformed the models with MobileNetV2, ResNet-50, and ResNet-101 in terms of impurity identification and segmentation accuracy. The *P* of Xception-65 impurity recognition segmentation was 95.03%, which was 1.31%, 5.12% and 4.25% higher than the values for MobileNetV2, ResNet-50, and ResNet-101, respectively.

For the identification and segmentation of grains or impurities, the DeepLabV3+ model with ResNet-50 achieved the highest *F*_1_ values of 83.63% and 87.18%, respectively. MobileNetV2, Xception-65, ResNet-50, and ResNet-101 obtained *F*_MIOU_ values of 0.6849, 0.7060, 0.7457, and 0.7074, respectively, as shown in Table 1. The *F*_MIOU_ values of ResNet-50 were 0.0608, 0.0397, and 0.0383 higher than those of MobileNetV2, Xception-65, ResNet-101, respectively.

In terms of speed, MobileNetV2 required about 234 ms to segment the grain and impurities in an image with a resolution of 512 pixels × 512 pixels; this was the fastest among the four backbones. There was no significant difference in speed between ResNet-50 and ResNet-101 at 256 ms and 261 ms, respectively. Xception-65 demonstrated the slowest image processing at 268 ms. Moreover, although ResNet-50 was not the fastest (256 ms/image), it was still the best choice considering *F*_1_ and *F*_MIOU_.

In terms of *F*_IOU_, the ResNet-50-based DeepLabV3+ model demonstrated better performance than the other three models, especially for the grain, as shown in Table 1. The *F*_IOU_ of model impurities trained by MobileNetV2, Xception-65, ResNet-50, and ResNet-101 were 0.7023, 0.7160, 0.7727, and 0.7221, respectively. The *F*_IOU_ value of ResNet-50 grain was 0.7186, which was 0.0512, 0.0441, and 0.0259 higher than those of MobileNetV2, Xception-65, and ResNet-101, respectively.

**Figure 6:**
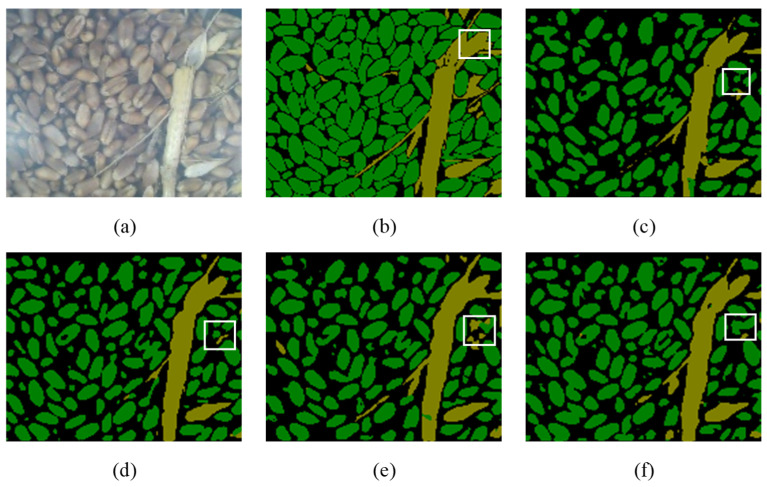
Different backbone network identification and segmentation effects. (**a**) Original image; (**b**) manually labeled image; (**c**) MobileNetV2; (**d**) Xception-65; (**e**) ResNet-50; (**f**) ResNet-101.

Visualizations of predicted images are shown in Figure 6c (MobileNetV2), Figure 6d (Xception-65), Figure 6e (ResNet-50), and Figure 6f (ResNet-101). The white boxed area in Figure 6 shows the difference in segmentation results using four backbones when segmenting impurities. MobileNetV2 and ResNet-101 only segmented some irrelevant background without any useful information. Xception-65 outperformed MobileNetV2 and ResNet-101, but still could not fully identify impurities. ResNet-50 outperformed other models in impurity identification and segmentation and could effectively identify and segment most impurities. Therefore, in this study ResNet-50 was selected as the backbone network of DeepLabV3+ to carry out the identification and segmentation of mechanically harvested wheat grain and impurity components. On this basis, the online detection of wheat impurity rate based on image information was realized.

### 3.2. ResNet-50 Online Recognition and Segmentation Effect Analysis

**Figure 7:**
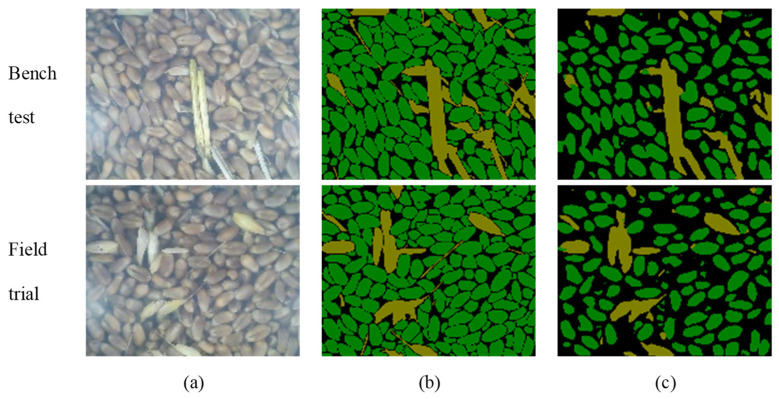
ResNet-50 recognizes segmentation effects online. (**a**) Original image; (**b**) manual annotation image; (**c**) device recognition segmentation image.

As shown in Figure 7, a detection image was randomly selected; the wheat grain and impurity components in the image were manually marked, and the recognition effects of the online detection device for wheat impurity rate during the bench test and field test were analyzed. Based on manual annotation, we found that ResNet-50 could segment most impurities such as straw and wheat husks. At the same time, the performance of ResNet-50 for the identification and segmentation of impurities was better than that for grain.

As shown in Table 2, in the bench test, the *P* value of the ResNet-50 model grain identification was 96.25%; the *R* value was 58.88%; the *F*_1_ value was 73.06%, and the *F*_IOU_ value was 0.5756. The *P* of the ResNet-50 model impurity identification was 93.40%; *R* was 69.73%; *F*_1_ was 75.37%; *F*_IOU_ was 0.6646, and the *F*_MIOU_ was 0.6201. In the field test, the *P* of the ResNet-50 model grain identification was 99.00%; the *R* was 51.62%; the *F*_1_ was 67.86%, and the *F*_IOU_ was 0.6646. The *P* of the ResNet-50 model impurity identification was 88.71%; *R* was 83.66%; *F*_1_ was 86.11%; *F*_IOU_ was 0.7561, and the *F*_MIOU_ was 0.7104. Whether it was a bench test or a field test, the model’s recognition and segmentation effects on impurities were better than that for grain.

### 3.3. Analysis of the Detection Effect of Impurity Rate

**Figure 8:**
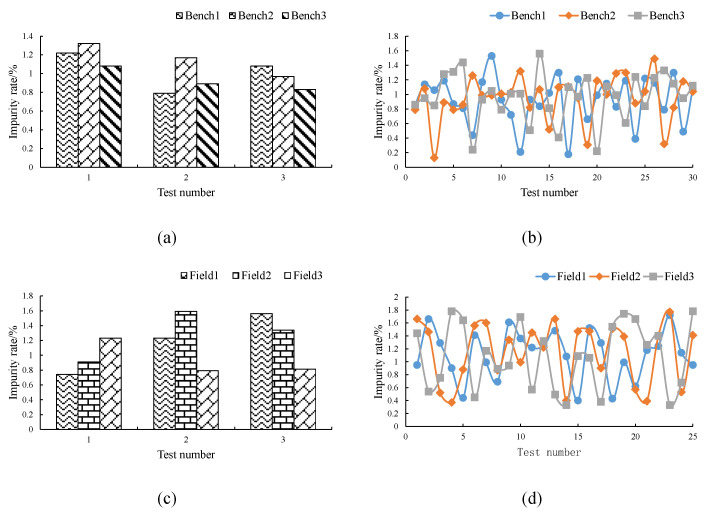
Detection results for the wheat impurity rate. (**a**) Manual test results from the bench test; (**b**) device test results from the bench test; (**c**) manual test results from the field test; (**d**) device test results from the field test.

During the test, the online monitoring device for wheat impurity rate based on the DeepLabV3+ model worked normally, realizing the dynamic online detection of wheat samples. The test results are shown in Figure 8 and Table 3.

During the bench test, the maximum value of the wheat impurity rate detected by the device was 1.56%; the minimum value was 0.13%, and the average value was 0.95%. The maximum value of the artificial detection of wheat impurity rate was 1.32%; the minimum value was 0.79%, and the average value was 1.04%. Compared with the manual detection results, the maximum absolute error of device detection was 0.2%, and the maximum relative error was 17.34%.

During the field test, the maximum value of the wheat impurity rate detected by the device was 1.78%; the minimum value was 0.33%, and the average value was 1.11%. The maximum value of the artificial detection of wheat impurity rate was 1.78%; the minimum value was 0.34%, and the average value was 1.12%. Compared with the manual detection results, the maximum absolute error of device detection was 0.06%, and the maximum relative error was 13.78%.

In the bench test, the maximum value of the coefficient of variation of the device detection results was 35.24%, and the minimum value was 31.79%; the maximum value of the coefficient of variation of the manual detection results was 17.39%, and the minimum value was 11.42%. In the field test, the maximum value of the coefficient of variation of the device detection results was 46.31%, and the minimum value was 33.43%; the maximum value of the coefficient of variation of the manual detection results was 28.63%, and the minimum value was 21.51%. Whether a field test or a bench test, the results from the device had significant fluctuation. This was mainly because the detection device calculated the real-time impurity rate by dynamically capturing the sample image and analyzing the single-layer image information. When the impurities in the image captured by the detection device occupied a large area, the value of the impurity rate in the detection result would be very large. When the impurities in the image captured by the detection device occupied a small area, the value of the impurity rate in the detection result would be relatively small.

Although there was a certain difference in the numerical values of the device and manual detection results, the results of the two detection methods showed that the impurity rate of the wheat in the actual operation process was less than 2%, and the impurity rate of wheat met the national standard. Therefore, the two detection methods were consistent in the qualitative identification of whether the wheat impurity rate met the national standard. It could be seen that the detection results of the device could objectively reflect the actual working conditions of the combine harvester, and the device provided technical support for the driver to grasp the working conditions of the combine harvester in real time.

## 4. Conclusions

In order to realize the online detection of wheat impurity content during mechanized harvesting, an online detection device for wheat impurity rate based on deep learning was designed. In this study, a segmentation model for mechanized harvesting wheat grain and impurity identification with four backbones was developed based on DeepLabV3+. The optimal backbone network was determined by indicators such as precision rate, recall rate, comprehensive evaluation index, intersection ratio, and average intersection ratio. On this basis, a quantitative model of wheat impurity content based on image information was established to realize the online detection of wheat impurity content. The results showed that the DeepLabV3+ model with ResNet-50 achieved the highest *F*_1_ on grain and impurity recognition segmentation at 83.63% and 87.18%, respectively. The *F*_MIOU_ value of ResNet-50 was 0.0608, 0.0397, and 0.0383 higher than those MobileNetV2, Xception-65, ResNet-101, respectively. Therefore, in this study, ResNet-50 was selected as the backbone network of DeepLabV3+ to carry out the identification and segmentation of mechanized harvesting wheat grain and impurity components. In terms of speed, it took about 256 ms for ResNet-50 to recognize an image with a segmentation resolution of 512 × 512 pixels using an Nvidia Quadro RTX5000 16 GB GPU. Based on the manual detection results, the maximum absolute error of the device detection during the bench test was 0.2%, and the maximum relative error was 17.34%; the maximum absolute error of the device detection during the field test was 0.06%, and the maximum relative error was 13.78%. Therefore, the mechanized wheat impurity rate detection model could meet the needs of actual production. This study could be used to help wheat farmers grasp the operating performance of a combine harvester in real time, thereby effectively improving the quality of mechanized wheat harvesting.

In the future, we will focus on the following work: First, we will collect images of different varieties of mechanically harvested wheat to enrich the content of the data set; The second is to improve the DeepLabV3+ network structure and reduce the problems of missing segmentation and excessive segmentation.

## Figures and Tables

**Table 1:** DeepLabV3+ model recognition and segmentation performance of different backbones.

Model Backbone		DeepLabV3+			
		MobileNetV2	Xception-65	ResNet-50	ResNet-101
Grains	*P* (%)	85.31	83.57	86.86	84.54
	*R* (%)	75.41	77.76	80.63	79.32
	*F*_1_ (%)	80.06	80.56	83.63	81.85
	*F*_IOU_	0.6674	0.6745	0.7186	0.6927
Impurities	*P* (%)	93.72	95.03	89.91	90.78
	*R* (%)	73.70	74.39	84.61	77.93
	*F*_1_ (%)	82.51	83.45	87.18	83.87
	*F*_IOU_	0.7023	0.7160	0.7727	0.7221
*F*_MIOU_		0.6849	0.7060	0.7457	0.7074
*I*_v_ (ms)		234	268	256	261

**Table 2:** Online recognition and segmentation performance of ResNet-50 model.

Index	Bench Test					Field Test				
	*P* (%)	*R* (%)	*F*_1_ (%)	*F*_IOU_	*F*_MIOU_	*P* (%)	*R* (%)	*F*_1_ (%)	*F*_IOU_	*F*_MIOU_
Grains	0.9625	0.5888	0.7306	0.5756	0.6201	0.9900	0.5162	0.6786	0.6646	0.7104
Impurities	0.9340	0.6973	0.7985	0.6646		0.8871	0.8366	0.8611	0.7561	

**Table 3:** Statistical data of the detection results of wheat impurity rate.

Test Type		$\bar{P_{\mathrm{Sz}}}(\%)$	$\bar{P_{\mathrm{Mz}}}(\%)$	*R*_Scv_ (%)	*R*_Mcv_(%)	*R*_az_ (%)	*R*_rz_ (%)
Bench test	1	0.92	1.03	35.24	17.39	0.11	10.68
	2	0.95	1.15	31.79	12.43	0.20	17.34
	3	0.97	0.93	33.00	11.42	0.04	4.29
Field test	1	1.11	1.18	33.43	28.63	0.06	5.1
	2	1.15	1.28	39.33	21.94	0.13	10.16
	3	1.08	0.94	46.31	21.51	0.13	13.78

## Data Availability

Not applicable.
